# Differential effects of sertraline in a predator exposure animal model of post-traumatic stress disorder

**DOI:** 10.3389/fnbeh.2014.00256

**Published:** 2014-07-30

**Authors:** C. Brad Wilson, Leslie D. McLaughlin, Philip J. Ebenezer, Anand R. Nair, Rahul Dange, Joseph G. Harre, Thomas L. Shaak, David M. Diamond, Joseph Francis

**Affiliations:** ^1^Comparative Biomedical Sciences, School of Veterinary Medicine, Louisiana State UniversityBaton Rouge, LA, USA; ^2^Pathobiological Sciences, School of Veterinary Medicine, Louisiana State UniversityBaton Rouge, LA, USA; ^3^Air Force Clinical Research Laboratory, Keesler Air Force BaseMS, USA; ^4^Medical Research Service, VA HospitalTampa, FL, USA; ^5^Departments of Psychology and Molecular Pharmacology and Physiology, Center for Preclinical and Clinical Research on PTSD, University of South FloridaTampa, FL, USA

**Keywords:** norepinephrine, serotonin, sertraline, PTSD rat, inflammation mediators, SSRI, elevated plus maze

## Abstract

Serotonin (5-HT), norepinephrine (NE), and other neurotransmitters are modulated in post-traumatic stress disorder (PTSD). In addition, pro-inflammatory cytokines (PIC) are elevated during the progression of the disorder. Currently, the only approved pharmacologic treatments for PTSD are the selective-serotonin reuptake inhibitors (SSRI) sertraline and paroxetine, but their efficacy in treating PTSD is marginal at best. In combat-related PTSD, SSRIs are of limited effectiveness. Thus, this study sought to analyze the effects of the SSRI sertraline on inflammation and neurotransmitter modulation via a predator exposure/psychosocial stress animal model of PTSD. We hypothesized that sertraline would diminish inflammatory components and increase 5-HT but might also affect levels of other neurotransmitters, particularly NE. PTSD-like effects were induced in male Sprague-Dawley rats (*n* = 6/group × 4 groups). The rats were secured in Plexiglas cylinders and placed in a cage with a cat for 1 h on days 1 and 11 of a 31-day stress regimen. PTSD rats were also subjected to psychosocial stress via daily cage cohort changes. At the conclusion of the stress regimen, treatment group animals were injected intraperitoneally (i.p.) with sertraline HCl at 10 mg/kg for 7 consecutive days, while controls received i.p. vehicle. The animals were subsequently sacrificed on day 8. Sertraline attenuated inflammatory markers and normalized 5-HT levels in the central nervous system (CNS). In contrast, sertraline produced elevations in NE in the CNS and systemic circulation of SSRI treated PTSD and control groups. This increase in NE suggests SSRIs produce a heightened noradrenergic response, which might elevate anxiety in a clinical setting.

## Introduction

Post-traumatic stress disorder (PTSD), an anxiety disorder recently reclassified as a trauma- and stressor-related disorder, can develop in response to real or perceived life-threatening situations. According to the Diagnostic and Statistical Manual of Mental Disorders 5 (DSM-5), a diagnosis of PTSD necessitates exposure to a traumatic event, intrusive recollections, avoidance of associated stimuli, negative cognitions/mood, hyperarousal, and a significant social impairment. All of these symptoms must persist for at least 30 days and not be due to illness, medication, or substance abuse (American, 2013). To date, no definitive diagnostic biomarkers have been identified for PTSD. Recent research, however, points toward physiological abnormalities in the hypothalamic-pituitary-adrenal (HPA) axis, sympathoadrenal medullary system, immune system, and neurotransmitters that may be implicated in the disorder (Liberzon et al., 1999; Söndergaard et al., 2004; Oosthuizen et al., 2005; Wilson et al., 2013, 2014a). Although neurotransmitters are modulated in PTSD development, it remains unclear whether serotonin (5-HT) is the only neurotransmitter affected by selective-serotonin reuptake inhibitors (SSRI). Changes in levels of other neurotransmitters might explain why SSRIs have met with such mixed results in PTSD therapy (Davidson et al., 2001; Watts et al., 2013). To evaluate the effects of sertraline on neurotransmitter modulation, we employed a well-documented predator exposure/psychosocial stress animal model of PTSD demonstrating three hallmark features of the disorder: hormonal abnormalities, a long-lasting traumatic memory, and persistent anxiety (Zoladz et al., 2008, 2012). This model also possesses both predictive and construct validity, making it sensitive to clinically effective pharmacologic agents while displaying similarities to human PTSD (Bourin et al., 2007).

5-HT is a neurotransmitter responsible for many functions in the central nervous system (CNS) and periphery. Serotonergic cell bodies originate primarily in the raphe nuclei, but every area of the CNS receives 5-HT innervation (McGeer et al., 1987). It influences aggression, arousal, sleep, anxiety, appetite, fear, learning, and other processes (Dubovsky, 1994). 5-HT is also the principle regulator of mood. A study by Peirson et al. (Peirson and Heuchert, 2000) found lower platelet 5-HT_2_ receptor function was associated with depressed mood, while Williams et al. (2006) demonstrated higher blood 5-HT levels were correlated with better mood. An increased mood and overall sense of well-being has been shown, in both psychiatric and physical disorders, as protective and positively correlated with resiliency behavior (Delamothe, 2005). Research has also demonstrated that 5-HT-uptake sites in platelets were lower in PTSD patients vs. controls (Arora et al., 1993). Lower 5-HT has also been implicated in diminished physical health. Muldoon et al. (2004) showed that a low prolactin response to fenfluramine, a drug that increases 5-HT levels, was associated with metabolic syndrome. Based on 5-HT’s action, it is reasonable to surmise SSRIs should be effective in PTSD. The SSRIs sertraline and paroxetine are the only Food and Drug Administration (FDA) approved drugs for PTSD, but the modulation of other neurotransmitters in response to 5-HT reuptake has yet to be clearly delineated.

Norepinephrine (NE) is a neurotransmitter involved in the regulation of psychiatric and physical processes (Zoladz and Diamond, 2013). In the brain, the locus coeruleus (LC) synthesizes and releases NE, which modulates multiple functions such as neuroplasticity, attention and memory, emotions, and psychological stress (Benarroch, 2009). The LC also has projections to the spinal cord where NE is released from postganglionic neurons in the sympathetic nervous system to initiate the “fight-or-flight” response. In addition, chromaffin cells in the adrenal medulla release NE and epinephrine into the bloodstream, increasing heart rate and blood flow to skeletal muscles and triggering the release of glucose. Persistent noradrenergic activity, however, has been linked with negative outcomes in patients with congestive heart failure (CHF; Francis et al., 1993) and diabetes (Ganguly et al., 1986). Studies have also shown that individuals with PTSD have elevated cerebrospinal fluid (CSF) levels of NE (Geracioti et al., 2001) and noradrenergic hyperresponsiveness to various stimuli (Liberzon et al., 1999). Moreover, dysregulation of noradrenergic neurons has been associated with hyperarousal and intrusive recollections attributable to PTSD (Southwick et al., 1999). A study by Bracha et al. (2005) noted irregularities in the number of cells of the LC in postmortem examinations of combat veterans diagnosed with PTSD. Thus, neurotransmitter modulation resulting in elevated NE levels might increase sympathetic drive and elevate anxiety.

Since the late 1980s, SSRIs have proven effective in the treatment of depression (Doogan and Caillard, 1992; Miller et al., 1998). In PTSD, however, SSRI efficacy can be classified as inconsistent at best (Friedman et al., 2007). A study by Davidson et al. (2001) which was a part of the FDA approval process for sertraline use in PTSD, demonstrated decreased severity of symptoms and an overall increase in functioning in the PTSD patients vs. controls. The results were achieved with multiple investigator- and self-rated assessments. This study, however, had uneven gender distribution (84% female), racial distribution (83% white), and traumatic event distribution (64% physical or sexual assault). The efficacy of sertraline in PTSD, therefore, may be variable due to gender, demographics, and/or type of incident. The data showed a 45% increase in symptom improvement in the treatment group, but also a 36% increase in symptom improvement in the placebo group. Taken together, these numbers indicate that the majority of the noted improvement may be due to a placebo effect. In addition, there were no physiological measures conducted to analyze actual neurotransmitter modulation during treatment. This information could be critical in determining the true efficacy of SSRIs, as neurotransmitter changes are not mutually exclusive events. With this in mind, this study sought to analyze the modulation of neurotransmitters and inflammatory components in the hippocampus, prefrontal cortex (PFC), CSF, and plasma after a 7-day sertraline treatment regimen.

## Materials and methods

### Ethics statement

This study was carried out in accordance with the recommendations of the Institute for Laboratory Animal Research’s 2011 *Guide for the Care and Use of Laboratory Animals*, under the auspices of an animal care and use protocol approved by the Louisiana State University Institutional Animal Care and Use Committee.

### Animals

Naïve adult male Sprague-Dawley rats (Harlan Laboratories, Indianapolis, IN) were used in all experiments. The rats were the same age (12 weeks) and approximately the same weight (±15 g) upon delivery. Rats were pair-housed in standard plastic microisolator cages with access to food and water *ad libitum*. The cages were maintained in ventilated racks and randomly assigned to a rack location to ensure groups were evenly distributed. The vivarium room was kept on a 12-h light/dark cycle (0700–1900), temperature was maintained at 20 ± 1°C, and humidity ranged from 23–42%. After a 1-week acclimation period, the mean weight of all rats was 308.5 g ± 2.5. Two cats, a male and a female (Harlan Laboratories, Indianapolis, IN) were used for all predator exposures. They were housed in an open room (15′ × 15′) in the vivarium with access to food, water, and enrichment devices *ad libitum*. The cat room was on the same light/dark cycle and maintained at a similar temperature and humidity.

### Stress induction

The predator exposure/psychosocial stress regimen is designed to induce a PTSD-like syndrome as true PTSD is clinically defined as a human disorder. Following the acclimation period, rats were weighed, ear-tagged, tail-marked, and 250–500 μL of blood was drawn from the tail vein. The rats were then randomly assigned to the PTSD or control group and returned to the vivarium for 24 h. The following day, PTSD rats were started on a predator exposure/psychosocial stress regimen. Briefly, PTSD rats were individually isolated in cylindrical, Plexiglas containers (IITC Life Science, Inc., Woodland Hills, CA) and canned cat food was smeared on the outside of the cylinders. The cylinders prevented direct contact with the cats, and the cat food induced movement in the cats. Rats were then placed in a stainless steel cage (76 cm × 76 cm × 60 cm) consisting of a solid metal floor with a hinged, metal rod door, with a cat for one hour. The first cat exposure was conducted during the light cycle (0700–1900). Ten days later, a second cat exposure was conducted during the dark cycle (1900–0700). In addition, the rats were subjected to psychosocial stress by changing their cage cohort daily. The predator exposure/psychosocial stress regimen continued for 31 days, after which certain PTSD and control group rats were administered sertraline intraperitoneally (i.p.) for 7 days. All groups were then euthanized via CO_2_ inhalation, blood was collected by intracardiac puncture, exsanguination via perfusion was conducted with a phosphate buffered solution, and the brains were removed. The hippocampus and PFC were dissected and flash-frozen in liquid nitrogen.

### Elevated Plus-Maze (EPM)

Rats were randomly selected for either the control or the PTSD group and were administered a baseline elevated plus-maze (EPM) prior to the predator exposure. The EPM was also administered after the 31-day stress regimen and again after the 7-day sertraline treatment. Rats were placed in the center of the EPM (EB-Instruments (Bioseb), Tampa Bay, FL) facing an open arm and allowed to roam freely for five minutes. Movement was monitored via an overhead camera and captured with a software program (BioEPM3C, EB-Instruments, Tampa Bay, FL). The primary measurements were the total number of arm entries and the time spent in the open vs. closed arms.

### Sertraline

Rats were pair-housed and treatment group animals were injected i.p. with sertraline HCl dissolved in 50% dimethyl sulfoxide (DMSO) and dH_2_O at 10 mg/kg for 7 consecutive days (Maj and Rogoz, 1999). Control rats were injected i.p. with vehicle.

### Real-time PCR analysis

Semi-quantitative real-time RT-PCR (*n* = 6/group) was used to determine the mRNA levels of interleukin-1β (IL-1β), interleukin-4 (IL-4), interleukin-10 (IL-10), and Toll-like receptor-4 (TLR4) in the PFC and hippocampus. Total RNA isolation, cDNA synthesis and RT-PCR were performed as previously described (Agarwal et al., 2011). Gene expression was measured by the ΔΔCT method and was normalized to GAPDH mRNA levels. The data is presented as fold change of the gene of interest relative to that of control animals.

### Western blot analysis

Tissue homogenates from the PFC and hippocampus were subjected to Western Blot (WB) analysis (*n* = 6/group) for the determination of protein levels of IL-1β, IL-4, IL-10, TLR4, and β-Actin. The extraction of protein and WB was performed as previously described (Agarwal et al., 2011). Primary antibodies were commercially obtained: IL-1β, and β-Actin, 1:1000 dilution (SC-7884 and SC-1616R respectively, Santa Cruz Biotechnology, Santa Cruz, CA), TLR4, IL-4, and IL-10, 1:1000 dilution (ab13556, ab9811, and ab9969 respectively, Abcam, Cambridge, MA). Secondary antibodies were commercially obtained: anti-rabbit, 1:5000 dilution (SC-2004, Santa Cruz Biotechnology, Santa Cruz, CA).

### High-Performance Liquid Chromatography (HPLC)

Neurotransmitter concentrations were detected using Eicom HTEC 500 HPLC system. The standard solutions of NE (MW 337.3), 5-HT (MW 212.68) and Isoproterenol (internal standard; MW 247.7) were each 1 ng/μL concentrations. Sample preparations were carried out as previously described (Agarwal et al., 2011; Wilson et al., 2014a).

#### HPLC detection of neurotransmitters

HPLC system working conditions: isocratic elution; mobile phase (Citrate buffer in methanol with EDTA and sodium octane sulfonate); Eicompak SC-3ODS (ID 3.0 × 100 mm) column; flow rate 340 μl/min; graphite working electrode WE-3G (Gasket GS-25), (+750 mV vs. Ag/AgCl electrode); temperature 25°C.

#### HPLC mobile phase

Citric acid monohydrate (8.84 g; MW 210.14), and 3.10 g of sodium acetate (MW 82.03) in 800 ml of MilliQ Ultrapure fresh water (>18.2 MΩ/cm) and 200 ml of HPLC grade methanol were added. EDTA (MW 372.24; 0.005 g) and sodium octane sulfonate (0.220 g), both from Dojindo Laboratories, Rockville, MD, were added.

### ELISA analysis

An ELISA kit was used to measure NE (MBS881383, MyBioSource, San Diego, CA) levels in the CSF and plasma according to manufacturer’s instructions.

### Statistical analysis

Data are presented as mean ± SEM. Statistical analysis conducted by one-way ANOVA with a Bonferroni *post hoc* test for multiple comparisons, unpaired Student’s *T*-tests for two-column analyses, and four-parameter logistic regression for curve fit. *p*-values less than 0.05 were considered significant. Statistical analyses were performed using Prism (GraphPad Software, Inc, La Jolla, CA; version 5.0).

## Results

### Elevated plus-maze performance

**Figure 1:**
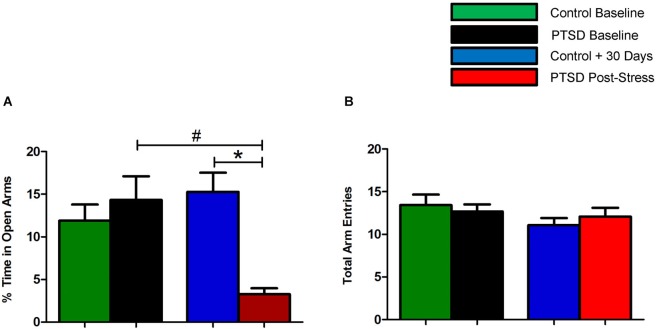
**Elevated plus-maze performance: within group measurements**. The PTSD group spent considerably less time in the open vs. closed arms compared to the control group and to baseline **(A)**. There were no differences noted in overall ambulations between or within groups **(B)**. All data are presented as mean ± SEM. *p < 0.05 relative to the control group. #*p* < 0.05 relative to within group measurements (baseline).

**Figure 2:**
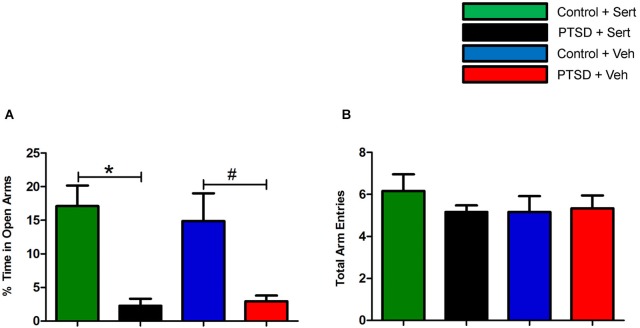
**Elevated plus-maze performance: between group measurements**. After sertraline treatment, the PTSD + Sert group demonstrated no measureable improvement vs. the control + Sert group, and the PTSD + Veh group displayed persistent anxiety vs. the control + Veh group **(A)**. There were no differences in overall ambulations in any of the four groups after the treatment period **(B)**. All data are presented as mean ± SEM. *p < 0.05 between the treatment groups. #*p* < 0.05 between the vehicle groups.

Prior to the start of the stress regimen, there were no differences noted in baseline open arm exploration (Figure 1A) or total arm entries (Figure 1B). After the 31-day stress regimen, the PTSD group spent considerably less time in the open vs. closed arms as compared to the control group, *t*_(22)_ = 5.10, *p* < 0.0001, and as compared to baseline, *t*_(22)_ = 3.86, *p* < 0.001 (Figure 1A). Overall ambulations, however, were not affected, *F*_(3,44)_ = 0.974, *p* > 0.05 (Figure 1B). After the 7-day sertraline treatment, the PTSD + Sert group displayed no increased open arm exploration vs. the PTSD + Veh group, *t*_(10)_ = 0.49, *p* > 0.05, or the control + Sert group, *t*_(10)_ = 4.59, *p* < 0.001. The control group also showed no difference between the control + Sert and control + Veh groups, *t*_(10)_ = 0.43, *p* > 0.05, (Figure 2A). No differences were found in overall ambulations between or within groups after the sertraline treatment, *F*_(3, 20)_ = 0.55, *p* > 0.05 (Figure 2B).

### CSF and plasma NE analysis

**Figure 3:**
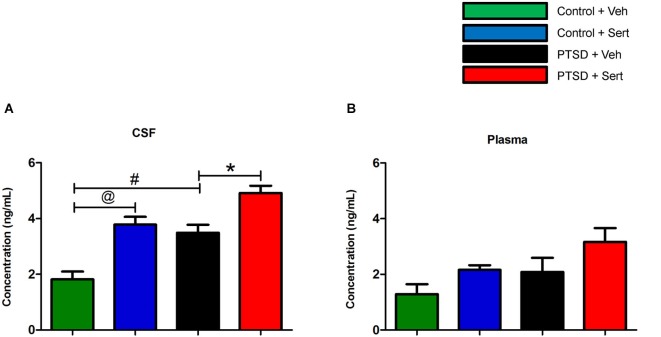
**CSF and plasma NE levels**. In the CSF, NE was elevated in the PTSD + Veh vs. the control + Veh group and in the control + Sert vs. the control + Veh group. NE was further elevated in the PTSD + Sert vs. the PTSD + Veh group **(A)**. In the plasma, NE was higher in the treatment groups, but it did not reach significance in any comparisons **(B)**. All data are presented as mean ± SEM. *p < 0.05 between the PTSD groups. @*p* < 0.05 between the control groups. #*p* < 0.05 between the vehicle groups.

In the CSF, NE was elevated in the PTSD + Veh vs. the control + Veh group, *t*_(8)_ = 4.22, *p* < 0.01. Sertraline raised NE levels in the control + Sert vs. the control + Veh group, *t*_(8)_ = 4.96, *p* < 0.02, and NE was further elevated in the PTSD + Sert vs. the PTSD + Veh group, *t*_(8)_ = 3.72, *p* < 0.01 (Figure 3A). In the plasma, NE was higher in the treatment groups, but it did not reach significance in any comparisons (Figure 3B).

### Inflammatory markers

**Figure 4:**
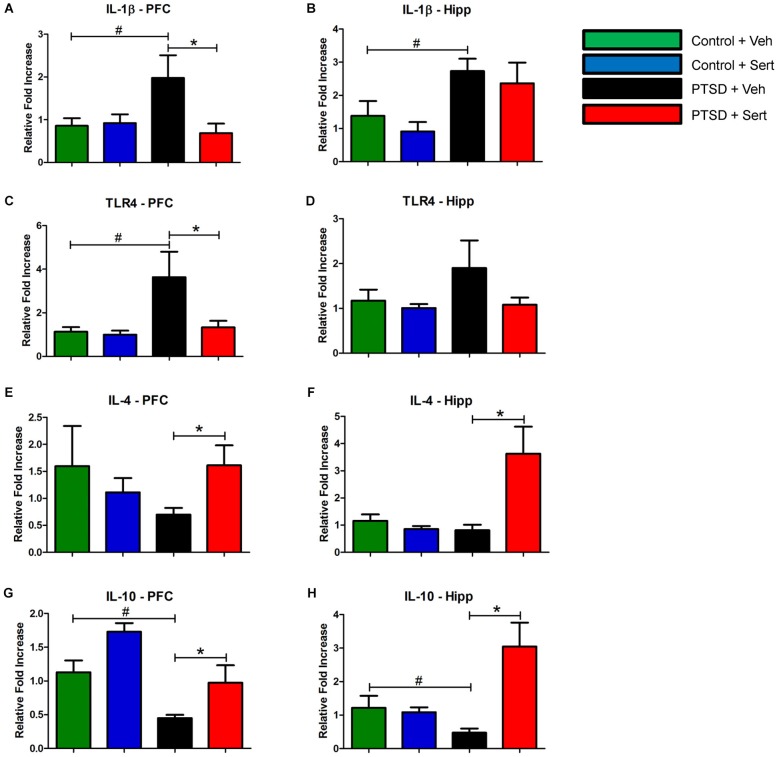
**Pro- and Anti-Inflammatory Marker mRNA**. In the PFC and hippocampus, the PTSD group demonstrated elevated mRNA levels of IL-1β (**A** and **B**) and TLR4 (**C** and **D**). Conversely, there were diminished levels of IL-4 (**E** and **F**) and IL-10 (**G** and **H**) in the same regions. Sertraline administration normalized the elevated PIC mRNA and up-regulated anti-inflammatory cytokine (AIC) to levels similar to or higher than the control + Veh group. All data are presented as mean ± SEM. *p < 0.05 between the PTSD groups. #*p* < 0.05 between the vehicle groups.

**Figure 5:**
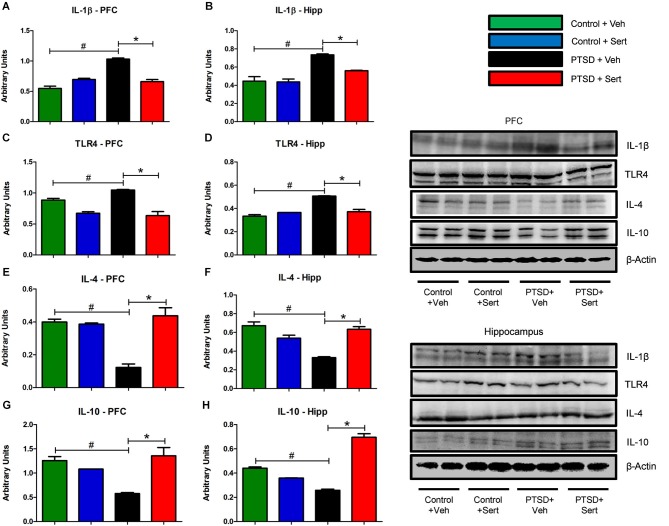
**Pro- and Anti-Inflammatory Marker Protein**. The PFC and hippocampus demonstrated elevated protein levels of IL-1β (**A** and **B**) and TLR4 (**C** and **D**) in the PTSD group. Conversely, the levels of AIC protein in these regions were lower for IL-4 (**E** and **F**) and IL-10 (**G** and **H**). Sertraline administration also normalized the aberrant protein to levels similar as the control + Veh group. All data are presented as mean ± SEM. *p < 0.05 between the PTSD groups. #*p* < 0.05 between the vehicle groups.

In the PFC and hippocampus, the PTSD group demonstrated elevated mRNA levels of IL-1β, *F*_(3,18)_ = 3.56, *p* < 0.05 and *F*_(3,20)_ = 3.53, *p* < 0.05 (Figures 4A,B) and TLR4, *F*_(3,20)_ = 4.11, *p* < 0.05 and *F*_(3,20)_ = 1.44, *p* > 0.05 (Figures 4C,D). Conversely, there were diminished levels of IL-4, *F*_(3,20)_ = 0.99, *p* > 0.05 and *F*_(3,20)_ = 6.65, *p* < 0.05 (Figures 4E,F) and IL-10, *F*_(3,20)_ = 9.57, *p* < 0.05 and *F*_(3,20)_ = 7.34, *p* < 0.05 (Figures 4G,H) in the same regions. Sertraline administration normalized the elevated pro-inflammatory cytokines (PIC) mRNA and up-regulated anti-inflammatory cytokine (AIC) to levels similar to or higher than the control + Veh group. The PTSD group also displayed elevated protein levels in the PFC and hippocampus of IL-1β, *F*_(3,4)_ = 53.04, *p* < 0.05 and *F*_(3,4)_ = 22.15, *p* < 0.05 (Figures 5A,B) and TLR4, *F*_(3,4)_ = 25.78, *p* < 0.05 and *F*_(3,4)_ = 43.14, *p* < 0.05 (Figures 5C,D). The levels of AIC protein were lower for IL-4, *F*_(3,4)_ = 25.59, *p* < 0.05 and *F*_(3,4)_ = 27.01, *p* < 0.05 (Figures 5E,F) and IL-10, *F*_(3,4)_ = 13.16, *p* < 0.05 and *F*_(3,4)_ = 134.10, *p* < 0.05 (Figures 5G,H). Sertraline administration also normalized the aberrant protein to levels similar as the control + Veh group.

### Neurotransmitter modulation

**Figure 6:**
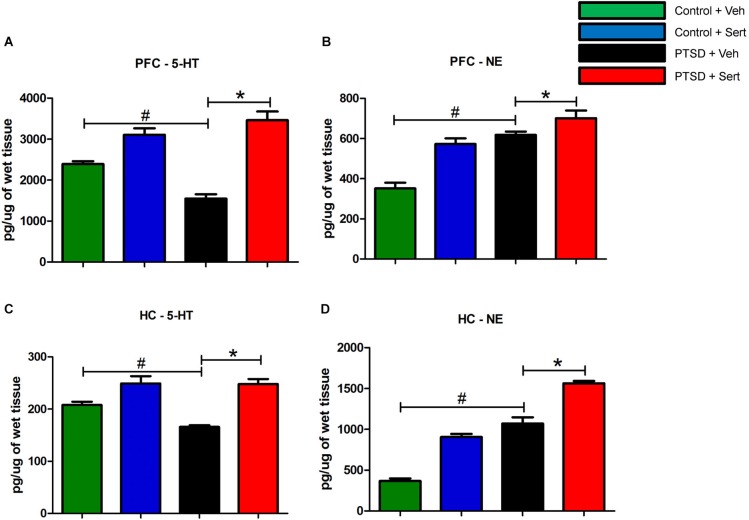
**5-HT and NE Modulation**. In the PFC, the level of 5-HT **(A)** was significantly lower in the PTSD + Veh vs. the control + Veh group. Conversely, the level of NE **(B)** was significantly higher in the PTSD + Veh group. Sertraline expectedly raised 5-HT levels in the PTSD + Sert and control + Sert groups to levels higher than the control + Veh group **(A)**, but it also elevated NE in the PTSD + Sert and control + Sert groups to levels higher than the control + Veh group **(B)**. In the hippocampus, 5-HT **(C)** was significantly lower in the PTSD + Veh vs. the control + Veh group, while NE **(D)** was higher. Similar to results in the PFC, sertraline normalized 5-HT to pre-stress levels, but it doubled NE in the control + Sert group and more than tripled NE in the PTSD + Sert group compared to the control + Veh group **(D)**. All data are presented as mean ± SEM. *p < 0.05 between the PTSD groups. #*p* < 0.05 between the vehicle groups.

To investigate the influence of sertraline on neurotransmitter modulation, we examined endogenous levels of biogenic amines in the hippocampus and PFC of control and PTSD animals using HPLC. In the PFC, the level of the tryptamine 5-HT (Figure 6A) was significantly lower in the PTSD + Veh vs. the control + Veh group, *t*_(10)_ = 6.64, *p* < 0.0001. Conversely, the level of the catecholamine NE (Figure 6B) was significantly higher in the PTSD + Veh group, *t*_(10)_ = 8.04, *p* < 0.0001. Sertraline expectedly raised 5-HT levels in the PTSD + Sert and control + Sert groups to levels higher than the control + Veh group *F*_(3,20)_ = 32.62, *p* < 0.0001 (Figure 6A), but it also elevated NE in the PTSD + Sert and control + Sert groups to levels higher than the control + Veh group *F*_(3,20)_ = 26.59, *p* < 0.0001 (Figure 6B). In the hippocampus, 5-HT (Figure 6C) was significantly lower in the PTSD + Veh vs. the control + Veh group, *t*_(10)_ = 6.03, *p* < 0.0001, while NE (Figure 6D) was higher, *t*_(10)_ = 8.94, *p* < 0.0001. Similar to results in the PFC, sertraline normalized 5-HT to pre-stress levels, *F*_(3,20)_ = 18.35, *p* < 0.0001 (Figure 6C), but it doubled NE in the control + Sert group and more than tripled NE in the PTSD + Sert group compared to the control + Veh group, *F*_(3,20)_ = 124.10, *p* < 0.0001 (Figure 6D).

## Discussion

The present study sought to analyze neurotransmitter modulation and pro- and AICs in the PFC, hippocampus, CSF, and plasma of rats subjected to a PTSD model and subsequently treated with sertraline. A myriad of animal models designed to create PTSD-like effects are reported, but the model by Zoladz et al. (2008, 2012) has been shown to cause common symptoms reported in humans with PTSD (Brewin et al., 2000; Nemeroff et al., 2006) such as heightened anxiety, exaggerated startle response, impaired cognition, and increased cardiovascular reactivity. Although animal models have certain well-understood limitations, a major component missing from human PTSD research is the ability to ascertain physiological data directly from specific brain regions immediately after a stressful event. The majority of the human physiological data gathered *in vivo* is derived from saliva, blood and urine, which may not accurately reflect neurotransmitter modulation in the brain and certainly cannot distinguish between changes in specific brain regions. We have successfully obtained such data with this Sprague-Dawley rat model, and to our knowledge, we are the first to report the modulation of biogenic amines and inflammatory components in response to sertraline administration in the brains of PTSD animals. Three novel and important findings emerged from this study. First, 5-HT suppression in the brain regions examined was normalized with sertraline, but NE levels also significantly increased in response to the treatment. Second, sertraline produced anti-inflammatory effects as evidenced by decreased PICs and elevated AICs. Lastly, despite attenuating inflammatory markers, sertraline provided no positive benefit in relation to anxiety or behavior.

The modulation of various neurotransmitters observed with the predator exposure/psychosocial stress model is in concert with many of the neurotransmitter changes seen in human PTSD patients (Yehuda et al., 1992; Arora et al., 1993; Geracioti et al., 2001, 2013). Previous research has shown that stress blocks long-term potentiation (LTP) in the hippocampus as well as impairs hippocampal function (Kim and Diamond, 2002; Diamond et al., 2007). The hippocampus, the primary region for spatial and long-term memory storage, expresses all of the 5-HT receptor families and reflects overall serotonergic functions relating to cognition and mood in this region (Berumen et al., 2012). During stress, glucocorticoid production can reduce the excitability of hippocampal neurons, and 5-HT may have a protective effect against such damage by activating 5-HT_1A_ receptors (Joca et al., 2007). Persistent activation of the HPA axis and excessive production of glucocorticoids, however, may directly reduce hippocampal 5-HT levels and adversely affect normal serotonergic transmission, thus contributing to heightened fear, depressed mood, and reduced resilience. The hippocampus also contains multiple NE receptors which, when activated during stress, may contribute to the reinforcement of long-term memories (Jurgens et al., 2005). In a study by Geracioti et al. involving male combat veterans with PTSD, CSF concentrations of NE were significantly higher vs. controls (Geracioti et al., 2001). This finding could possibly explain why memories formed during extremely stressful events persist over time. Other evidence of catecholamine dysregulation in PTSD includes elevated urine catecholamine excretion, exaggerated biochemical responses to yohimbe, and clinical efficacy of adrenergic blockers (Southwick et al., 1999).

The PFC is responsible for executive functions such as consequences, drive, and social “control”. It is highly innervated by serotonergic neurons from the raphe nuclei, and it expresses an abundance of 5-HT receptors. The serotonergic neurons and 5-HT receptors, specifically the 5-HT_1A_ and 5-HT_2A_ receptors, are key modulators of the PFC-amygdala-corticolimbic circuit involved in threat and emotional responses (Fisher et al., 2011). PTSD-related aberrancies in this serotonergic system may cause inappropriate or incomplete extinction of conditioned fear. The PFC also contains NE receptors and receives input from NE neurons from the LC, which are activated during the stress response (Finlay et al., 1995). Pathogical or stress-related elevations of NE in the PFC, however, may inhibit working memory and performance (Zhang et al., 2013). Current neuroimaging research indicates that the PFC is hyporesponsive during symptomatic PTSD states and that this responsiveness is inversely proportional to symptom severity (Shin et al., 2006). Whether marked elevations in NE directly or indirectly diminish PFC responsiveness and subsequent performance on cognitive emotional tasks remains unclear.

The role of inflammation in pathological conditions such as cardiovascular disease, diabetes mellitus, metabolic syndrome, and neurological disease is well established (Pall and Satterlee, 2001; Elks and Francis, 2010; Agarwal et al., 2011; Alexopoulos et al., 2013). We recently demonstrated oxidative stress and inflammation were up-regulated in the brain and systemic circulation of rats subjected to the predator exposure model (Wilson et al., 2013). In chronic stress-related conditions such as PTSD, a sustained sympathoexcitatory state can alter the T_H_1/T_H_2 cell balance and increase PIC production (Chrousos, 2000). Research presents strong evidence that cytokines and inflammation may be directly linked to psychiatric disorders, but whether they are causal or nonspecific immunological side-effects remains unresolved (Schiepers et al., 2005). Inflammatory cytokine levels have been shown to be inversely proportional to 5-HT levels, and it is hypothesized that PICs can diminish tryptophan by activating the tryptophan-metabolizing enzyme indoleamine-2,3-dioxygenase (IDO; Heyes et al., 1992). There is also evidence that PICs counteract the negative feedback of glucocorticoids on the HPA axis, altering its function (Miller et al., 1999). In our previous work, we found that valproic acid (VA) attenuated inflammation, but it differed from sertraline in that it did not result in noradrenergic hyperresponsiveness and actually modulated NE to levels similar to untreated controls. In addition, VA lowered anxiety and resulted in vast improvements on the EPM (Wilson et al., 2014b). The fact that both of these compounds decreased inflammatory components but did not equally improve EPM performance indicates inflammation and oxidative stress may be contributors, but not the sole causal factors in terms of PTSD pathophysiology.

We have demonstrated that sertraline increases 5-HT and NE, and that it attenuates inflammation in the hippocampus, PFC, and CSF. Based on these mechanisms, its administration should result in decreased anxiety and improved resilience. In our experiments, however, we observed no improvement on the EPM that indicated reduced anxiety in the rats. The EPM is widely used as a measure to test fear or anxiety and has been extensively validated for use in rats (Pellow et al., 1985; Korte and De Boer, 2003). Anxiogenic compounds or procedures can increase avoidance of the fear-provoking open arms, whereas anxiolytic compounds or procedures can increase open arm exploration (Pellow et al., 1985). It should be noted, however, that not all anxiolytic compounds modify behavior in PTSD. Zoladz et al. (2013) demonstrated the ineffectiveness of clonidine and amitriptyline, but showed a marked improvement in behavior with tianeptine. The anxiolytic effects of increased 5-HT and attenuated inflammation should have resulted in increased open arm exploration compared to the PTSD + Veh group. The fact that no significant changes were noted between the treated and untreated PTSD groups suggests other mechanisms might be acting as endogenous anxiogenic agents. One such mechanism might be elevated CNS NE and increased sympathetic tone. Noradrenergic hyperresponsiveness has previously been shown to contribute to PTSD pathophysiology (Southwick et al., 1993). It has also been suggested that the LC neurons, responsible for CNS NE production, constitute the first or second step of the PTSD circuit (Bracha et al., 2005). Our findings of elevated NE in the hippocampus, PFC, and CSF despite sertraline administration provide sound evidence that exaggerated sympathoexcitation may be a primary reason underlying the modest efficacy of SSRIs in PTSD.

## Conclusions

We utilized a predator exposure/psychosocial stress animal model of PTSD to analyze the effects of sertraline on neurotransmitter modulation and inflammation in the rat hippocampus, PFC, CSF, and plasma. We found that sertraline increased 5-HT and NE levels in the hippocampus, PFC, and CSF. We also discovered that sertraline attenuated inflammation by lowering PICs and elevating AICs. Despite these seemingly beneficial changes, however, anxiety did not diminish in the PTSD + Sert vs. the PTSD + Veh groups. We propose that noradrenergic hyperresponsiveness, evident by exaggerated levels of NE present in the hippocampus, PFC, and CSF, might be a primary factor in persistent anxiety and a major reason that SSRIs have demonstrated poor efficacy in PTSD. To our knowledge, this is the first study to provide a molecular rationale for this unsatisfactory record of accomplishment. This insight might allow for more targeted pharmacologic therapies with an emphasis on inflammatory suppression and control of sympathetic drive. It would be an oversimplification, nonetheless, to presume that a persistent noradrenergic tone is the sole causal factor in PTSD development. The autonomic nervous system (ANS), endocrine system, and immune system are indelibly linked with CNS disorders and identifying one system as the cause of PTSD might be impossible. Overall, our results demonstrate that there are CNS-specific modifications in neurotransmitters, immunomodulators, and ANS activity in response to sertraline in the predator exposure/psychosocial stress model. Further research is critical to delineate, if possible, which of these systems actually contributes to PTSD pathophysiology and which are producing nonspecific responses common to multiple psychiatric etiologies. In addition, direct comparisons with SSRIs and other compounds demonstrating effectiveness in PTSD such as VA and tianeptine are warranted.

## Funding and disclosures

Funding for this study was via LSU SVM corp grants and the Clinical Research Laboratory, 81st Medical Support Squadron, Keesler Air Force Base, MS. David Diamond was supported by a Career Scientist Award from the Veterans Affairs Department. The opinions expressed in this paper are those of the authors and not of the Department of Veterans Affairs, the Department of Defense, the US Air Force, or the US government.

## Conflict of interest statement

The authors declare that the research was conducted in the absence of any commercial or financial relationships that could be construed as a potential conflict of interest.
